# Decoding host–microbiome interactions through integrative multi-omics and artificial intelligence: from biological mechanisms to predictive modelling

**DOI:** 10.3389/frmbi.2026.1906484

**Published:** 2026-09-28

**Authors:** Gayatri Krishna S., Narchikundil Reeja, Shemmy Sadanandan, Bhagya Mappurath, Akash Chatterjee, Ginny Antony, Tony Grace

**Affiliations:** 1 Department of Genomic Science, School of Biological Sciences, Central University of Kerala, Kasaragod, Kerala, India; 2 Department of Plant Science, School of Biological Sciences, Central University of Kerala, Kasaragod, Kerala, India

**Keywords:** artificial intelligence, deep learning, host-microbiome, machine learning, multi-omics

## Abstract

The mutual communication between the human host and its microbiome plays a fundamental role in maintaining metabolic and immunological homeostasis. Disruptions in this delicate ecosystem are heavily linked to severe pathologies like inflammatory bowel disease (IBD), metabolic syndromes, and cancer. Whilst traditional single-omics approaches offer limited taxonomic snapshots, multi-omics strategies are bridging the gap by mapping the complex, multi-layered networks driving host-microbe interactions. However, integrating these highly dimensional, heterogeneous datasets creates significant computational bottlenecks. Artificial Intelligence (AI), spanning machine learning and deep learning architectures, has emerged as a critical catalyst to resolve this crisis. AI transforms complex data into predictive models for early diagnosis, clinical biomarker discovery, and personalised therapeutics. Despite persistent hurdles in model generalisation and data standardisation, the convergence of multi-omics and AI is actively driving the future of precision medicine.

## 1 Introduction

The human body is not an island, but a complex, dynamic ecosystem. Over the past two decades, research has radically shifted our understanding of human biology as a symbiotic assembly of the eukaryotic host and trillions of co-evolving microbial cells (Reynoso-García et al., 2022). This complex ecosystem, collectively known as the microbiota, encodes millions of unique genes that vastly expand the host’s metabolic and immunological repertoire. The intricate, bidirectional communication between the host and its microbiome plays a fundamental role in maintaining homeostasis, regulating metabolic pathways, tuning the immune system, and even modulating neural signalling via the gut-brain axis. Consequently, disruptions in these homeostatic interactions, also termed dysbiosis, have been mechanistically linked to a wide variety of pathologies, including inflammatory bowel disease (IBD), metabolic syndromes, neurodegenerative disorders, and cancer (Banfi et al., 2021).

Historically, our understanding of these microbial communities relied on single-omics approaches. Targeted 16S rRNA gene sequencing provided essential taxonomic snapshots, answering which microorganisms are present in a given community, whilst shotgun metagenomics expanded this to clarify the genomic potential of the environment. However, single-omics studies possess inherent, systemic limitations. It cannot determine which genes are actively transcribed (metatranscriptomics), which proteins are translated (metaproteomics), or which biochemical phenotypes are ultimately realised (metabolomics). Biological processes are heavily regulated across multiple layers, and relying on a single omics layer offers an incomplete and often misleading view of the host-microbe interface (Arıkan and Muth, 2023).

To overcome these limitations, the field has rapidly moved towards systems-level approaches. By concurrently capturing data from both the microbe and the host, researchers can finally begin to map the multi-layered causal networks that drive health and disease. Yet, the transition to multi-omics integration has introduced a monumental computational bottleneck. Multi-omics datasets are characterised by extreme high-dimensionality (tens of thousands of features mapped across relatively small patient cohorts), inherent sparsity, high noise-to-signal ratios, and severe batch effects introduced by cross-platform variability. Traditional, conventional statistical frameworks, such as linear regressions or standard pairwise correlations, are fundamentally insufficient to handle these architectures. They struggle with non-linear biological dependencies, fail to scale efficiently, and are often blind to the emergent properties inherent in multi-layered biological networks (Eckhardt et al., 2024).

This computational crisis is precisely why Artificial Intelligence (AI) has emerged as an indispensable catalyst for modern microbiome research. Machine learning (ML) and deep learning (DL) architectures are uniquely suited to digest multi-dimensional, heterogeneous data. Advanced AI frameworks, ranging from Random Forests and Gradient Boosting to Convolutional Neural Networks (CNNs) and Autoencoders, excel at automated feature extraction, pattern recognition, and uncovering latent, non-linear relationships across disparate data types. AI does not merely manage the noise of multi-omics data; it transforms static data points into predictive, actionable models, accelerating breakthroughs in antimicrobial peptide discovery, functional gene annotation, and precision therapeutics.

## 2 The biological basis of host-microbiome interactions

The human body is home to a structured biological community consisting of bacteria, fungi, archaea, and various other microbes. Preserving a stable equilibrium within this microscopic ecosystem is essential for human health, whereas dysbiosis, a disruption in this microbial balance, can foster the overgrowth of harmful organisms. This disruption can either act as a direct driver of illness or contribute to disease progression independently of those opportunistic microbes. Consequently, medical research has spent decades investigating how these microscopic communities safeguard human health, with numerous studies highlighting their distinct influences on non-communicable ailments like Inflammatory Bowel Disease (IBD) (Zhou and Zhao, 2025).

### 2.1 Gut-brain axis

The last three decades have revealed the evidence for a complex relationship between enteric microbiota and brain function, and the involvement of the microbiome in the gut-brain axis in the pathophysiology and treatment of several brain disorders (Costa et al., 2024). The gut–brain axis is a bidirectional communication system linking the gut, gut microbiota, and central nervous system (CNS) through neural, immune, endocrine, and metabolic signalling pathways (Cryan et al., 2019). Multi-omics analysis in ASD revealed that host genetic defects linked to intestinal barrier dysfunction were associated with microbial dysbiosis, reduced level of butyrate-producing bacteria (Zhong et al., 2026). These findings support the involvement of the gut–brain axis by demonstrating how host genetic factors, gut microbial communities, and microbial metabolites collectively contribute to neurodevelopmental outcomes.

### 2.2 Metabolic crosstalk

A fundamental mechanism through which the gut microbiota influences host physiology is by producing bioactive metabolites that regulate immune, metabolic, and neuroendocrine pathways. Amongst the most well-characterised metabolic axes are the short-chain fatty acid (SCFA) pathway, the bile acid (BA) pathway, and the tryptophan (Trp) catabolism pathway. Each pathway produces distinct classes of molecules that interact with specific host receptors, transcription factors, and immune effectors, collectively shaping metabolic homeostasis, immune tone, and neuroendocrine function.

#### 2.2.1 Short-chain fatty acids

Short-chain fatty acids (SCFAs) are a group of small monocarboxylic acids that are synthesised by intestinal microbiota through the anaerobic breakdown of non-digestible dietary carbohydrates in the large intestine (Koh et al., 2016). The major SCFAs produced during this process are acetate, propionate, and butyrate, which collectively contribute to host metabolism, immune regulation, and gut homeostasis (Akhtar et al., 2021). Recent advances in integrative multi-omics approaches have significantly improved the understanding of SCFA-mediated host-microbiome interactions. Using a multi-omics approach, Xu et al. (2026) showed that SCFAs produced by the maternal gut microbiota, together with progesterone, play an important role in determining offspring birth weight in cows, suggesting that the maternal microbiome contributes to fetal developmental programming through metabolite-mediated hormonal interactions. This highlights how multi-omics approaches are expanding the known biological scope of SCFA signalling beyond the intestinal environment.

#### 2.2.2 Bile acid metabolism

Bile acids are cholesterol-derived molecules synthesised in the liver via the classical CYP7A1-mediated pathway and the alternative CYP27A1-mediated pathway, yields cholic acid (CA) and chenodeoxycholic acid (CDCA) in humans and are released into the intestine. The gut microbiota plays a crucial role in bile acid metabolism by converting primary bile acids into secondary bile acids through enzymatic reactions such as deconjugation, dihydroxylation and oxidation. These microbially modified bile acids function as signalling molecules involved in lipid metabolism, glucose homeostasis, and immune regulation (Fogelson et al., 2023). Integrative metabolomic and metagenomic analyses have revealed complex microbial networks regulating bile acid transformation and host receptor signalling pathways, including FXR and TGR5 (Wahlstrom et al., 2016).

#### 2.2.3 Tryptophan catabolism

Tryptophan (Trp) is an essential aromatic amino acid composed of a β-carbon connected to the 3rd position of an indole group. Of the 20 common canonical amino acids, Trp is the largest by molecular weight. Although Trp is the least abundant amino acid in protein and cells, it is a biosynthetic precursor of a large number of microbial and host metabolites (Alkhalaf and Ryan, 2015). It is required for protein synthesis and for maintaining metabolic and immune homeostasis (Zheng et al., 2025). Tryptophan serves as a precursor for the synthesis of serotonin (5-HT) (Liu et al., 2021), a key neurotransmitter involved in the regulation of mood, sleep, and appetite, as well as melatonin, a hormone derived from serotonin that controls the circadian sleep–wake cycle (Höglund et al., 2019).

Trp metabolism follows three major pathways in the gastrointestinal tract: (1) the direct transformation of Trp into several molecules, including ligands of the aryl hydrocarbon receptor (AhR), by the gut microbiota; (2) the kynurenine pathway (KP) in both immune and epithelial cells via indoleamine 2,3-dioxygenase (IDO)and (3) the serotonin (5-hydroxytryptamine [5-HT]) production pathway in enterochromaffin cells via Tryptophan hydroxylase 1 (TPH1) (Agus et al., 2018). The balance amongst these pathways serves as a sensitive indicator of gut immune activation and microbial status, and its dysregulation is now mechanistically linked to IBD, neuropsychiatric conditions, and systemic metabolic disorders (Xia and Huang, 2025).

### 2.3 Host genetic influence

The gut microbiota consists of a diverse community of bacteria, archaea, fungi, protozoa, and viruses that collectively contribute to human health and the development of disease (Thursby and Juge, 2017; Kho and Lal, 2018; Wu et al., 2021). Research has demonstrated that host genetics can significantly influence the composition of the gut microbiota (Cahana and Iraqi, 2020; Qin et al., 2022). Large-scale twin studies, genome-wide association studies (GWAS), multi-omics integration frameworks, and the integration of artificial intelligence (AI) have progressively revealed how specific host single nucleotide polymorphisms (SNPs) govern microbial colonisation and how the microbiome reprograms host gene expression through epigenetic mechanisms.

#### 2.3.1 Host SNPs affecting microbiome composition

GWAS have successfully identified genetic loci associated with various human traits and diseases (Abdellaoui et al., 2023). Similarly, microbiome GWAS (mGWAS) aims to identify host genetic polymorphisms that interact with the composition and abundance of the gut microbiota. mGWAS have identified significant loci and their connection to bacterial taxa, highlighting the influence of host genetics on microbiome composition in health and disease (Awany et al., 2018). Studies in the Japanese population (Ortega-Reyes et al., 2025) and in European populations (Davenport et al., 2015; Lopera-Maya et al., 2022) showed that variations in host genes can affect the abundance of specific gut microbes, suggesting an important role of host genetics in shaping the gut microbiome.

#### 2.3.2 Epigenetic modulation

Epigenetic modulation refers to heritable changes in gene expression that occur without alterations in the underlying DNA sequence (Bird, 2007). Major epigenetic mechanisms include DNA methylation, histone modifications, and regulation by non-coding RNAs (Jaenisch and Bird, 2003). These mechanisms play a critical role in controlling immune responses, metabolism, and cellular homeostasis (Allis and Jenuwein, 2016). Emerging evidence suggests that the gut microbiome can influence host epigenetic patterns through the production of microbial metabolites and other signalling molecules, thereby affecting gene expression and physiological functions (Qin and Wade, 2018). Together, these processes constitute a bidirectional ‘epigenome–microbiome axis’ in which microbial metabolites reprogram host chromatin, whilst host epigenetic states in turn influence the mucosal environment and microbial community composition (Pepke et al., 2024).

Microbial metabolites can influence host epigenetic regulation by modulating DNA methylation patterns. Short-chain fatty acids (SCFAs), particularly butyrate and propionate, regulate the activity of DNA methyltransferases (DNMTs) and methylation of genes involved in immune responses and metabolic homeostasis. In addition, microbiota-derived folate, methionine, and B vitamins contribute to the S-adenosylmethionine (SAM) pool required for DNA and histone methylation, thereby linking microbial metabolism to host epigenetic regulation (Woo and Alenghat, 2022).

Additionally, microbially derived folate, methionine, and other B vitamins contribute to the S-adenosylmethionine (SAM) methyl donor pool that fuels DNMT- and histone methyltransferase-catalyzed methylation reactions, providing a metabolic link between microbial biosynthetic capacity and host epigenetic regulation (Pepke et al., 2024).

Non-coding RNAs (ncRNAs), including microRNAs (miRNAs), long non-coding RNAs (lncRNAs), and small interfering RNAs (siRNAs), represent an emerging bidirectional communication channel between host and microbiome. Host-derived faecal miRNAs have been shown to enter bacterial cells and selectively modulate microbial gene expression and growth, shaping gut microbial community composition through a cross-kingdom RNA-based mechanism. Conversely, microbial signals modulate host miRNA expression profiles in intestinal epithelial cells and immune cells, altering post-transcriptional gene regulatory networks that govern barrier function and inflammatory tone. Long non-coding RNAs serve as chromatin scaffolds and transcriptional co-regulators, and their expression in intestinal epithelial and immune cells is profoundly shaped by microbial colonisation status, as demonstrated by dramatic lncRNA expression differences between germ-free and conventionally colonised animal intestines (Zheng et al., 2025).

## 3 Multi-omics technologies for studying host–microbiome interactions

Multi-omics is an integrated biological strategy that simultaneously analyses multiple categories of molecular data from a biological system, such as genomic, transcriptomic, proteomic, metabolomic, and other “omics” technologies. By combining these heterogenous data sets, researchers are able to elucidate complex molecular interactions, identify disease-associated biomarkers, and gain deeper insights into the mechanisms underlying health and disease. Such integrative analysis contributes significantly to the advancement of precision medicine by identifying novel biomarkers, enhances the understanding of disease mechanisms and supports the development of personalised therapeutic interventions (Turanli et al., 2018). Furthermore, the convergence of multi-omics technologies with artificial intelligence (AI) is transforming biological research by enabling the efficient analysis of large and complex datasets. AI-driven approaches can uncover hidden patterns, predict biological outcomes, and accelerate discoveries in precision medicine and systems biology (Yetgin, 2025).

Currently, several omics approaches are available for the analysis of biological datasets, such as metagenomic and metataxonomic sequencing, which identify microbial species and their genetic presence in the samples. Metaproteomics reveals the protein content and indicates the functionality of the community. Metabolomics unveils the small molecule composition identifying metabolites that can originate from the host, microbes, or diet. Amongst these, small molecules with immunomodulatory potential, such as short-chain fatty acids and bile acids, play significant roles in IBD (Zhang et al., 2024).

### 3.1 Metagenomics

Metagenomic approaches have been used to characterise various niches for decades, from marine environments to toxic soils to arthropod disease vectors to the human microbiome Chiu and Miller (2019); Mathieu et al. (2023). It is a culture independent tool used for studying microbial communities in the environment through DNA extraction and sequencing technologies (Kumari and Rai, 2020). Metataxonomic sequencing, also known as 16s rRNA sequencing approach or amplicon sequencing technique, is one of the most widely used sequencing approaches in metagenomics. This targeted approach employs primers that target specific conserved regions of the hypervariable loop within bacterial ribosomal RNA genes, followed by PCR amplification. Whilst this approach is primarily designed for profiling bacterial communities, it can also be used to study eukaryotic microorganisms by targeting conserved genetic markers such as the internal transcribed spacer (ITS1 and ITS2) regions, the 18S ribosomal RNA gene, or the D1/D2 domains of the 26S ribosomal RNA gene. The effectiveness of amplicon sequencing is largely supported by the availability of extensive reference databases, which facilitate the identification and classification of microbial taxa (Chiu and Miller 2019; Fernández-Carro et al., 2025).

Despite its widespread application, amplicon sequencing has several limitations. Its dependence on conserved target regions can restrict taxonomic resolution, making accurate species-level identification challenging in some cases. Additionally, PCR amplification may introduce biases and artifacts, including the formation of chimeric sequences, which can affect taxonomic assignments and compromise data quality (Fernández-Carro et al., 2025). To overcome these limitations, Whole Metagenome Sequencing (WMS) has emerged as a different technique to analyse the whole genome of a single microbe present in the sample. Unlike amplicon sequencing, WMS sequences all DNA present within a sample, including both microbial and host genetic material. This comprehensive strategy enables the detection of a broader range of microorganisms and provides greater taxonomic and functional resolution, often identifying all microbes, including rare and abundant taxa, than amplicon-based methods at comparable sequencing depths (Purushothaman et al., 2022; Navgire et al., 2022).

The high cost and analytical complexity are the main limitations of whole metagenome sequencing (WMS). In the case of WMS, it sequences all DNA within the sample, generating large datasets that demand substantial computational resources and bioinformatics expertise. The presence of host DNA further complicates analysis, as host-derived sequences must often be removed before microbial profiling can be performed. In addition, obtaining sufficient sequencing depth may be difficult in samples with low microbial abundance. However, continuous improvements in sequencing platforms and data analysis tools are helping to overcome these challenges (Fernández-Carro et al., 2025).

### 3.2 Metatranscriptomics

Metatranscriptomics involves the large-scale analysis of RNA molecules to characterise the transcriptional activity of microbial communities. Unlike metagenomics, which reveals the genetic potential of a microbiome, metatranscriptomics provides a more detailed view of the actively expressed genes and metabolic functions of microorganisms under specific environmental conditions. By identifying transcriptionally active microbial taxa and their functional pathways, this approach offers valuable insights into the dynamic behaviour of microbial communities and their interactions with the host (Fernández-Carro et al., 2025; Gao et al., 2023).

However, RNA is inherently less stable than DNA and is more susceptible to degradation, making sample collection, handling, and preservation critical for obtaining reliable metatranscriptomic data (Stark et al., 2014). Similar to metagenomic studies, metatranscriptomic analyses are often challenged by the predominance of host-derived sequences, which can complicate the detection of microbial transcripts. Nevertheless, a major advantage of metatranscriptomics is its ability to simultaneously understand gene expression profiles from both the microbiome and the host, providing a comprehensive understanding of biologically relevant host–microbe interactions (Gao et al., 2023).

Recent studies have highlighted the importance of host RNA molecules, particularly non-coding RNAs (ncRNAs) such as microRNAs (miRNAs) and circular RNAs (circRNAs), in mediating host responses to the gut microbiome and influencing disease phenotypes. Integrating metatranscriptomic and host transcriptomic datasets with RNA–RNA interaction resources can therefore provide valuable insights into the regulatory mechanisms governing immune responses and inflammation in disorders such as inflammatory bowel disease (IBD) (Zhang et al., 2024).

More broadly, transcriptomic analyses offer direct information about cell- and tissue-specific gene expression patterns. These analyses enable the identification and quantification of transcripts, assessment of alternative splicing events and protein isoform diversity, and evaluation of the effects of genetic variation on gene expression through approaches such as expression quantitative trait loci (eQTL) mapping and allele-specific expression (ASE) analysis. Collectively, these insights are essential for understanding cellular and tissue functions and for elucidating how alterations in gene expression contribute to health, disease development, and physiological adaptation (Manzoni et al., 2018).

### 3.3 Metaproteomics

Metaproteomics is a high-throughput approach that focuses on the identification and quantification of proteins produced by microbial communities within complex biological samples. Analysing expressed protein directly, it provides valuable insights into the functional activities of microorganisms in their native environments (Shrestha et al., 2021). In host-associated ecosystems such as the human gut, metaproteomics allows the simultaneous assessment of both microbial and host proteins, offering a comprehensive understanding of the molecular interactions that influence immune function, epithelial barrier integrity, and host–microbiome balance. Recent advancements in mass spectrometry technologies and bioinformatics tools have significantly improved protein detection and quantification, enabling researchers to explore taxon-specific activities as well as functional abundance across diverse microbial populations (Fernández-Carro et al., 2025).

Microbial proteins play a crucial role in regulating host signalling pathways and contribute to various physiological processes, including immune modulation, maintenance of cellular homeostasis, and preservation of intestinal barrier integrity (Zhang et al., 2024). In the case of healthy individuals, the gut epithelium is protected by a mucin layer that acts as a physical barrier, which minimises direct interactions between microbial and host proteins (Sheng and Hasnain, 2022). However, in conditions such as inflammatory bowel disease (IBD), inflammation and disruption of the mucosal barrier can permit microbial proteins to access host tissues and interact with host proteins, potentially influencing cellular signalling networks and disease progression (Zhou et al., 2022). But the information regarding microbial and host protein interaction is limited and required further in-depth studies.

To address this knowledge gap, integrative protein- protein interactions (PPIs) resources have emerged as valuable tools for exploring the functional interplay between microbial and host proteins. By combining metaproteomic and host proteomic data, these resources can help predict how microbial proteins affect host cellular pathways and contribute to disease-associated processes. However, the widespread application of such approaches is often constrained by the technical complexity, substantial sample requirements, and high costs associated with generating large-scale proteomic datasets. Consequently, alternative strategies that integrate metataxonomic and host transcriptomic data are increasingly being adopted. Metataxonomic analyses can identify microbial species enriched in specific disease conditions, and their corresponding proteomes can be obtained from databases such as UniProt. At the same time, host transcriptomic profiles can be used to find protein expression patterns, enabling the prediction of potential host–microbial PPIs and providing deeper insights into the molecular mechanisms underlying disease onset and progression (Zhang et al., 2024).

### 3.4 Metabolomics

Metabolomics focuses on the study of the metabolome, the complete set of small molecules which are produced during cellular metabolic processes of the host and its associated microbiota (Lee and Banerjee, 2020). These tiny molecules are responsible for the immune homeostasis, energy production, and signal transmission of the host. Key microbial metabolites, including short-chain fatty acids, bile acids, and tryptophan-derived compounds, have been shown to influence various physiological and pathological processes (Tang et al., 2025). Metabolite -protein association that integrates the metabolomics and proteomics data, which provides a better understanding of these interactions.

There are mainly three approaches to carry out metabolite extractions, such as untargeted analysis, semi-targeted analysis and targeted analysis. In untargeted screening, a large number of unknown features are profiled, allowing researchers to identify relative differences in metabolite abundance between conditions or across populations. This approach is an exploratory study to find out particularly novel metabolites associated with genetic disorders, disease states, or engineered metabolic pathways. In contrast, in semi-targeted metabolomics innumerable molecules were precisely identified and quantified; this approach enables researchers to generate comprehensive datasets that would otherwise require hundreds of individual biochemical assays, allowing for the detailed characterisation of biological networks, pathways, and their functional interactions. Although the semi-targeted studies are often hypothesis-generating rather than hypothesis-driven. The targeted metabolomics approaches are designed to address specific biological hypotheses and often provide deeper and more quantitative insights, with absolute quantification allowing accurate measurement of molecular concentrations and biological relevance (Liu et al., 2025).

However, the vast chemical diversity of metabolites, their frequent transient interactions with proteins and the current limitations of metabolomics technologies pose major challenges for their full characterisation. Improved annotation frameworks and multi-omics integration strategies can help to understand these complex interactions and improve our understanding of their roles in different biological conditions and diseases (Zhang et al., 2024).

Understanding complex biological systems requires the integration of multiple omics datasets, as individual omics layers provide only a partial view of the molecular processes occurring within an organism. By combining data from genomics, transcriptomics, proteomics, metabolomics, and metagenomics, researchers can gain a more comprehensive understanding of the biomolecules, pathways, and regulatory mechanisms involved in host–microbe interactions. To facilitate such analyses, numerous computational tools have been developed to investigate host–microbial relationships. These tools are generally classified into three major categories based on their prediction strategies: sequence-based, structure-based, and machine-learning-based approaches (Zhang et al., 2024). Each category utilises distinct methodologies to integrate multi-omics data and uncover the mechanisms through which the gut microbiome influences host physiology and disease processes.

Sequence-based methods primarily rely on protein sequence information to predict potential interactions between microbial and host proteins. By integrating metaproteomic and host proteomic datasets, these approaches identify sequence similarities, conserved domains, motifs, and interaction signatures that may indicate biologically relevant protein–protein interactions. Such analyses provide valuable insights into the molecular mechanisms by which microbial proteins modulate host cellular pathways and contribute to health and disease (Tanca et al., 2016).

An overview of the principal multi-omics techniques applied to gut microbiome research is presented in **Table 1**, alongside a summary of their integration with artificial intelligence–based approaches and the key limitations that constrain each method. This synthesis serves as a technical reference point for the discussion that follows, illustrating the trade-offs researchers must navigate when combining heterogeneous omics layers with computational modelling.

**Table 1:** Multi-omics techniques and their key limitations.

Omics technique		Approach	Key limitation	Reference
Metagenomics	16S/amplicon sequencing	Taxonomic profiling using conserved microbial marker regions	Limited taxonomic resolution; difficult in species-level identification; PCR can cause bias, Artifacts and chimeric sequences	Fernández-Carro et al. (2025)
	Whole metagenome sequencing (WMS)	Provides broader taxonomic and functional resolution than amplicon sequencing	High cost, large datasets, substantial computational requirements and host-DNA contamination; Sufficient sequencing depth can be difficult when microbial abundance is low	Purushothaman et al. (2022); Navgire et al. (2022)
Metatranscriptomics		Captures transcriptionally active microbial communities and expressed genes	Host-derived RNA can dominate samples, making microbial transcripts difficult to detect	Stark et al. (2014)
Metaproteomics	Metaproteomics/host–microbial proteomics	Enables investigation of microbial and host proteins and their potential effects on immune and epithelial functions	Information about direct microbial–host protein interactions remains limited; Disruption of the mucosal barrier may complicate interpretation	Zhang et al. (2024); Sheng and Hasnain (2022); Zhou et al. (2022)
	Integrated metaproteomics/PPI analysis	Uses protein–protein interaction resources to predict functional host–microbe interactions	Large-scale proteomics requires substantial sample quantities, technical expertise and high costs	Zhang et al. (2024)
Metabolomics	Targeted Metabolomics	Hypothesis-driven measurement of predefined metabolites; Enables accurate and absolute quantification	Limited to known and predefines metabolites; Usually lower coverage than untargeted approach	Tang et al. (2025); Lee and Banerjee (2020)
	Semi-targeted Metabolomics	Provides identification and quantification of numerous metabolites simultaneously offering a broad coverage and annotation than targeted approaches	Semi-targeted approaches can remain hypothesis-generating rather than hypothesis-driven; Less comprehensive than untargeted and less quantitatively rigorous than fully targeted analysis	Liu et al. (2025)
	Untargeted Metabolomics	Exploratory profiling of metabolites without preselecting specific compounds; Useful for discovering novel biomarkers, disease associated metabolites and metabolic alterations	Many identified features remain unidentified; Provides relative quantification rather than absolute.	Liu et al., 2025
Multi-omics + AI		Integration of heterogeneous omics datasets and AI can reveal complex biological patterns and potential biomarkers	Large, heterogeneous dataset s increase analytical complexity and require sophisticated computational approaches	Turanli et al. (2018); Yetgin (2025)

### 3.5 Challenges in multi-omics data integration

Multi-omics data integration aims to harmonise multiple layers of biological data, such as epigenomics, transcriptomics, proteomics, and metabolomics (Zhang et al., 2024). As a rapidly evolving field, multi-omics integration combines data from diverse molecular layers to provide a holistic understanding of complex biological systems, facilitating the discovery of cross-layer interactions, revealing disease-associated mechanisms, and identifying clinically relevant pathways that support advancements in health diagnostics, precision medicine, and therapeutic development. However, several challenges are evident when merging varied omics data sets and methodologies, interpreting vast data dimensions, streamlining longitudinal sampling and analysis, and addressing the ethical implications of managing sensitive health information (Subramanian et al., 2020; Mohr et al., 2024). Multi-omics data originates from various technologies, resulting in heterogeneity between studies, leading to its own unique noise, detection limits, bias in data interpretation and missing values. Advanced computational tools and statistical methods are required to handle the high dimensionality and complex relationships within multi-omics data, moving beyond simple correlation methods. Despite these challenges, the field is evolving with novel strategies, such as ML algorithms, which can handle data heterogeneity and high dimensionality leading to a more comprehensive understanding of HMIs in IBD. Initiatives and open-access platforms are increasing for sharing multi-omics datasets and promoting data standardisation (Zhang et al., 2024).

Multi-omics data integration faces several major challenges; including the lack of standardised preprocessing protocols across different omics datasets (genomics, transcriptomics, proteomics, metabolomics, etc.) creates difficulties in the harmonisation of results because each data type has unique structures, distributions, measurement errors, and batch effects (Zito, 2025). In addition, the analysis of large and heterogeneous multi-omics datasets requires advanced expertise in bioinformatics, statistics, machine learning, programming, and biology, making it a significant bottleneck for many researchers. Also, selecting the most appropriate integration method is challenging because available approaches differ substantially in their underlying algorithms, assumptions, and applications. Finally, interpreting integrated results and translating them into biologically meaningful insights remains difficult due to model complexity, incomplete functional annotations, and the risk of drawing misleading conclusions (Picard et al., 2021).

In order to overcome these challenges, a wide range of advanced multi-omics integration approaches has been developed. Techniques like Similarity Network Fusion (SNF) allow the acquisition of complementary biological information across datasets by integrating sample similarity networks derived from different omics layers into a united graphical representation (Alif and Zhang, 2025). A Bayesian latent factor mode, Multi-Omics Factor Analysis (MOFA), is used to uncover the principal sources of variation within and between datasets, separating shared biological signals from those specific to individual omics platforms. On the other hand, the common patterns across different datasets can be identified using a tool like Multiple Co-inertia Analysis (MCIA) by maximising covariance and projecting them into a shared dimensional space (Meng et al., 2014). Thus, these unsupervised approaches integrate datasets without requiring predefined phenotypic labels. so they are commonly used for exploratory studies, finding shared or omics specific patterns. However these approaches are not guided by a predefined outcome, the resulting factors, clusters or components may not necessarily correspond to clinically meaningful phenotypes and require subsequent biological or clinical validation. In contrast, supervised approaches such as DIABLO, is an integrative framework that leverages predefined phenotypic groups to identify correlated features across datasets whilst selecting key biomarkers associated with the phenotype of interest (Singh et al., 2019).

Although multi omics integration techniques have the capability to handle high dimensional datasets, their scalability varies depending according to the underlying computational framework. Network based approaches such as SNF require construction and iterative fusion of similarity networks, whereas latent factor approaches like MOFA reduces complex datasets into smaller numbers of factors. When there are substantially more number of molecular variables than samples, supervised frameworks like DIABLO may become computationally and statistically difficult. Consequently, scalability of each method should be evaluated not only in terms of computational requirements but also in terms of model stability and the risk of overfitting increases dimensionally. Unlike these methodological frameworks, an integrated code-free platform like Omics Playground and Omics Notebook are also available for visualisation and exploratory analysis. These approaches incorporate interactive visualisations and guided workflows, which enables to overcome the current challenges of omics dataset integration by enhancing the reliability of biological interpretations and accelerate the discovery of meaningful insights from complex multi-omics datasets (Zito, 2025, Hayes et al., 2024).

Multi-omics integration has the potential to transform healthcare by enabling more precise and personalised approaches to disease diagnosis, prevention, and treatment. Combining information from multiple biological layers, it provides a comprehensive understanding of disease mechanisms and individual variability. However, the successful implementation of multi-omics in clinical practice depends on the development of standardised analytical pipelines, advanced computational methods, robust validation of findings, secure data storage and sharing systems, and effective incorporation into existing healthcare frameworks. Looking ahead, the convergence of multi-omics with artificial intelligence, digital twin technologies, and other emerging innovations is expected to drive the next generation of precision medicine, enabling more accurate predictions, personalised interventions, and improved patient outcomes (Mohr et al., 2024).

## 4 Artificial intelligence in host–microbiome research

Computational modelling has long served as a vital tool for analysing microbiome datasets, mapping interspecies dynamics, and tracking community interactions. This mathematical foundation has progressed into sophisticated machine learning methodologies, most notably deep learning, a core branch of artificial intelligence. Today, specialised frameworks such as Convolutional Neural Networks (CNNs), Recurrent Neural Networks (RNNs), and Long Short-Term Memory (LSTM) networks are being widely implemented to study complex microbial datasets. LSTMs are particularly advantageous because they utilise distinct gating architectures that prevent gradients from vanishing or exploding, thereby drastically improving the model’s capacity to recognise long-term dependencies and subtle biological trends within sequential microbiome data (Zhou and Zhao, 2025). Yet much of this literature, including the machine learning and deep-learning applications reviewed below, remains predominantly comparative and descriptive rather than mechanistically explanatory. Rigorous head-to-head benchmarking of methods across shared datasets, preprocessing pipelines, and evaluation criteria is still uncommon in the field, which makes it difficult to draw general conclusions about which approach is genuinely superior for a given task (Papoutsoglou et al., 2023).

AI-driven analyses of microbiome data have yielded significant advances in microbial profiling and functional prediction, phenotypic characterisation, clinical biomarker discovery, patient segmentation strategies, and distinction of disease variants (Probul et al., 2024).

In the study published by Monteiro et al. (2024), artificial intelligence was comprehensively integrated into host-microbiome research to overcome the computational barriers associated with high-dimensional, count-compositional sequencing data. The researchers implemented domain-specific feature engineering to extract 2,194 biological variables across multiple taxonomic ranks, formatting the data into counts, relative abundances, and centred log ratio transformations to capture subtle microbial patterns. To mitigate severe dataset multicollinearity, a regularised Ridge regression framework optimised with a 10-fold cross-validation feature selection approach was deployed. This predictive modelling successfully quantified the host-microbiome interactome, revealing that the ruminal population accounts for 36% of the variation in host residual feed intake (RFI) whilst simultaneously mapping phenotypic variances in milk fat and protein efficiencies. Furthermore, the study introduced a novel AI driven ensemble and network analysis that cross-examined multiple differential abundance methods, providing a robust, noise-reduced mechanism to accurately classify animal efficiency status and isolate specific microbial biomarkers. This effect size, whilst genuinely informative, warrants some methodological framing: the 36% figure (R^2^ = 0.36) was obtained through 10-fold cross-validation within a single cohort of 454 genotyped Holstein cows and has not been replicated in an independent herd, breed, or feeding system. It is also worth noting explicitly that this is a veterinary application in dairy cattle feed efficiency rather than a human clinical study; it illustrates the analytical approach well but should not be generalised, as some of the broader AI microbiome literature tends to do, into a claim about precision-medicine-ready performance in humans.

But still, there are many challenges that AI faces when dealing with microbiome data, including, compositional data characteristics necessitating log ratio transformations, ultra-high dimensional feature space requiring robust feature selection, batch effect correction and sequencing depth normalisation, zero-inflation dynamics inherent to below-detection-limit observations, and multi-omics data harmonisation to integrate disparate biological layers (Probul et al., 2024). Notably, few of the individual studies discussed in this section address more than one or two of these challenges within the same analytical pipeline, and a standardised benchmarking protocol that jointly evaluates compositionality, dimensionality, batch effects, and zero-inflation together, rather than treating each as a separate methodological footnote, is still largely absent from the field (Papoutsoglou et al., 2023).

### 4.1 Machine learning for microbiome profiling

The human microbiome represents a complex, dynamic ecosystem deeply linked to host physiology, playing critical roles in metabolic homeostasis, immune system regulation, and the pathogenesis of diverse diseases such as diabetes, inflammatory bowel disease, and colorectal cancer. As high-throughput sequencing technologies (such as 16S rRNA gene amplicon and shotgun metagenomic sequencing) continue to mature, researchers are faced with generating massive datasets characterised by extreme multi-dimensionality, interpersonal variation, high sparsity, and zero-inflated distributions. Traditional univariate statistical methods typically fall short in analysing these datasets because they assess the protective or risk effects of individual bacterial populations in isolation, completely overlooking the dense web of ecological dependencies and microbial interactions. To overcome these constraints, supervised machine learning (ML) has emerged as a transformative computational paradigm in microbiome research. Instead of examining single organisms, ML algorithms process relative abundance profiles concurrently, treating combinations of operational taxonomic units (OTUs) or amplicon sequence variants (ASVs) as functional, collective biomarkers capable of learning complex structural patterns to differentiate disease states from healthy baselines (Topçuoğlu et al., 2020).

Despite the widespread adoption of ML in predicting host traits, the field has historically grappled with methodological vulnerabilities, such as data leakage, lack of rigorous data splitting, over-optimistic performance reporting, and an implicit bias towards overly complex “black-box” configurations that sacrifice biological interpretation (Papoutsoglou et al., 2023).

#### 4.1.1 Random forest

In microbiome host trait prediction, Random Forest functions as an ensemble decision tree method that operates by generating a vast collection of individual, decorrelated decision trees during the training phase (Zhou and Gallins, 2019). Decorrelation amongst trees is achieved not only through bootstrap resampling of samples, but critically, through random subsampling of the candidate OTUs considered at each node split; this feature-level randomisation, rather than bootstrapping alone, is what most effectively prevents trees from converging on the same dominant taxa in high-dimensional, redundant microbiome data, and is a distinction often glossed over in applied microbiome papers (Zhou and Gallins, 2019). To handle the structural challenges of microbiome data, such as high sparsity and when the number of Operational Taxonomic Units (OTUs) vastly outnumbers the samples, Random Forest utilises bootstrapping to train each tree on a random subset of data (Zhou and Gallins, 2019). The final phenotypic or trait prediction is determined by averaging the continuous numeric predictions across all bootstrap trees or through modal voting for categorical classifications (Zhou and Gallins, 2019). This random sub-sampling protects the model from severe overfitting, a common issue given the extensive noise inherent to high-throughput genetic sequencing (Zhou and Gallins, 2019). Furthermore, because it does not rely on traditional linear assumptions, Random Forest excels at capturing complex, multi-microbe interactive patterns without requiring the data to conform to rigid multivariate normal distributions.

Flemer et al. (2018) developed a Random Forest (RF) classifier trained on log-transformed OTU abundance profiles derived from oral swabs and faecal samples to distinguish CRC-positive individuals from healthy controls across a cohort of 234 subjects. The RF model achieved 96% specificity using oral microbiota alone, which improved substantially upon integration of faecal microbiota data, raising sensitivity to 76% for CRC and 88% for polyps, demonstrating the enhanced predictive power of multi-source microbiome data fusion. The classifier further identified differentially abundant taxa, including *Streptococcus* and *Prevotella* spp., as discriminatory features, illustrating the capacity of machine learning to extract biologically meaningful biomarkers from high-dimensional compositional microbiome data. Supporting analytical approaches, including Principal Coordinates Analysis (PCoA), co-abundance group analysis, and bacterial network mapping, complemented the RF classifier by elucidating structural relationships between oral and colonic microbial communities in CRC. This study exemplifies the translational potential of AI-driven microbiome analysis, demonstrating how machine learning can convert complex microbial abundance data into clinically actionable, non-invasive disease predictions.

Several methodological caveats temper how far these results should be extrapolated. The Flemer et al. (2018) cohort (99 CRC cases, 32 polyps, and 103 controls; n = 234 in total) is modest by classification-modelling standards relative to the dimensionality of OTU-level data. The oral-only model’s headline 96% specificity corresponded to an AUC of 0.893 and, importantly, a considerably lower sensitivity of only 53% for CRC (67% for polyps); reporting the post-integration sensitivity figures (76%/88%) without this baseline risks overstating what the oral-microbiota-alone classifier actually achieves. Furthermore, the model was evaluated within the original study cohort rather than validated in an external or prospective population, so its performance under real-world screening conditions remains unestablished rather than merely “clinically actionable” as sometimes implied in secondary literature.

Beyond this specific case study, Random Forest’s practical limitations in microbiome applications deserve more emphasis than the sequential method descriptions typically give them. Performance is sensitive to hyperparameters such as the number of trees and the number of features sampled at each split; feature-importance rankings can be unstable across resampling runs when correlated taxa are present, complicating biomarker prioritisation; and although RF is comparatively robust to overfitting relative to a single decision tree, it still requires nested cross-validation, keeping feature selection and model evaluation on separate data folds, to avoid the optimistic performance bias that arises when both steps are performed on the same dataset, a distinction the applied microbiome literature does not always observe (Papoutsoglou et al., 2023).

#### 4.1.2 Support vector machines

Support Vector Machines act as a non-parametric, linear-category classifier optimised to locate an optimal separating decision boundary within a complex feature space (Zhou and Gallins, 2019). Specifically, SVM aims to construct a hyperplane in a high-dimensional mathematical space that maximises the geometric distance, or “margin,” between the closest sample points of opposing trait classes, which are known as the support vectors (Zhou and Gallins, 2019). When host phenotypic traits cannot be cleanly separated using linear boundaries due to overlapping microbial abundance profiles, non-linear variations of SVM are applied (Zhou and Gallins, 2019). These variations deploy specialised kernel similarity functions to project the low-dimensional OTU count matrices into higher-dimensional spaces where structural patterns become mathematically separable (Zhou and Gallins, 2019). By relying on only a subset of critical data points (the support vectors) rather than the entire distribution, SVM offers a robust and mathematically rigorous framework that reduces the risk of structural overfitting when analysing complex human or environmental disease states.

In practice, however, SVM’s performance on microbiome data is considerably less consistent than the theoretical description above might suggest. In a direct comparative benchmark across multiple disease datasets, linear SVM performed close to chance level on one of three tested cohorts and generally underperformed Random Forest and gradient boosting overall (Zhou and Gallins, 2019) — a finding worth stating plainly, since SVM is frequently presented in review sections as a broadly reliable option without this caveat. SVM also scales poorly with sample size, with training complexity that grows roughly quadratically to cubically with the number of samples; it offers limited native interpretability once a non-linear kernel is introduced, since the mapping into higher-dimensional feature space obscures which original taxa drive classification; and its performance is highly sensitive to kernel choice and hyperparameter tuning (e.g., the regularisation parameter C and kernel width or degree), decisions that are inconsistently reported and justified across the microbiome machine learning literature, undermining reproducibility.

#### 4.1.3 Gradient boosting

Gradient Boosting models represent a sequential ensemble methodology that constructs a predictive framework by iteratively correcting the mistakes of weak base learners (Zhou and Gallins, 2019). Unlike Random Forest models, which train independent trees in parallel and average their outputs, boosted tree algorithms train successive decision trees where each new model is explicitly fitted to the residuals or errors left behind by the preceding sequence of trees (Zhou and Gallins, 2019). Because tree-based configurations do not necessarily force terminal nodes to extend down to individual observations, they rely on modal or mean values inside those final nodes to fine-tune continuous or binary predictions (Zhou and Gallins, 2019). This optimisation approach allows gradient boosting to prioritise the highly complex, subtle variations in rare microbial taxa that are frequently overlooked by simpler algorithms, making it highly effective at refining the exact classification boundaries needed to predict intricate host clinical traits and disease phenotypes.

These advantages come with trade-offs that are rarely discussed alongside the method’s strengths. Gradient boosting has a considerably larger hyperparameter space than Random Forest, learning rate, tree depth, number of boosting rounds, and subsampling fraction all interact, which makes it more prone to overfitting on the small sample, high dimensional datasets typical of microbiome studies unless regularisation and early stopping are carefully tuned and validated on held-out data. Its sequential tree building process is also inherently less parallelisable than Random Forest’s, increasing computational cost for large feature sets such as ASV or OTU level tables. Finally, as with Random Forest, gradient boosting’s feature-importance scores can be biased towards taxa with many possible split points (i.e., more granular abundance values), which does not necessarily correspond to genuine biological importance and should be interpreted cautiously rather than taken directly as a ranked biomarker list.

### 4.2 Deep learning approaches

#### 4.2.1 Convolutional neural networks

In their methodological study, Reiman et al. (2020) present PopPhy-CNN, a specialised convolutional neural network (CNN) architecture designed to predict human host phenotypes, such as specific health or disease states, directly from gut microbiome taxonomic profiles. The primary challenge in applied metagenomics lies in the inherent structural limitations of standard operational taxonomic unit (OTU) abundance vectors (Reiman et al., 2020). Conventional predictive algorithms, including Support Vector Machines (SVMs) and Random Forests, treat distinct microbial species as independent variables (Reiman et al., 2020). By processing taxonomic data in an arbitrary, flat order, these traditional machine learning pipelines discard crucial phylogenetic configurations, overlooking the evolutionary relationships and co-dependent biological functions shared amongst closely related bacterial clades (Monshizadeh and Ye, 2024; Reiman et al., 2020).

To resolve this limitation and effectively exploit the advanced pattern recognition capabilities of deep neural networks, the authors engineered a spatial embedding protocol that reformats high-dimensional, sparse metagenomic profiles into two-dimensional matrices (Przymus et al., 2025; Reiman et al., 2020). The pipeline constructs a populated taxonomic tree template for each biological sample, where the quantitative relative abundance value of individual terminal species is recursively summed to propagate abundance scores upward through the internal parental nodes of the phylogenetic hierarchy (Reiman et al., 2020). This annotated tree structure is subsequently mapped into a 2D matrix by placing the root taxon at the top-left coordinate and populating successive rows sequentially with child nodes in the specific order dictated by their ancestral branching lineages (Reiman et al., 2020). This translation strategy ensures that evolutionary cousins and biologically similar microbes are positioned in immediate spatial proximity, effectively mirroring the property of spatial locality that defines pixel structures in standard image processing (Reiman et al., 2020).

By transforming abstract microbial data into a dense, visually structured domain, PopPhy-CNN leverages localised convolutional filters to scan across the taxonomic matrix, automatically extracting high-order, non-linear relationships amongst interacting microbial groups (Przymus et al., 2025; Reiman et al., 2020). Rather than evaluating isolated species, the sliding kernels of the convolutional layers detect complex, co-dependent biological features, such as the collective over- or under-representation of specific evolutionary groups, that correlate with system-wide changes in the host gut ecosystem (Reiman et al., 2020). Benchmarked across multiple diverse datasets for binary and multi-class clinical classification, this tree-embedded framework demonstrated robust predictive capacity and notable resilience against the overfitting issues typically triggered by high-dimensional datasets with small sample sizes (Reiman et al., 2020).

Crucially, the authors addressed the historical limitation of deep learning models operating as uninterpretable “black boxes” by integrating a backwards-tracking feature-extraction mechanism within the network architecture (Monshizadeh and Ye, 2024; Reiman et al., 2020). This interpretive framework computes distinct importance scores for each taxonomic node, permitting researchers to systematically isolate the specific groups of microbes driving the final phenotype prediction (Reiman et al., 2020). By successfully bridging high-accuracy classification with explicit biomarker discovery, PopPhy-CNN offers an advanced bioinformatics pipeline that clarifies how localised dysbiosis within complex microbial populations drives systemic disease manifestation in the human host (Reiman et al., 2020).

Notwithstanding these strengths, the original PopPhy-CNN evaluation was benchmarked on metagenomic datasets of moderate size, and, as with the Random Forest and SVM applications discussed above, reported predictive performance was assessed primarily through cross-validation within those datasets rather than in fully independent, prospectively collected cohorts (Reiman et al., 2020). The node-level importance scores generated by the interpretive framework identify taxa that are statistically associated with the model’s predictions; this is a meaningful step towards interpretability, but it is not equivalent to establishing a causal or mechanistic role for those taxa in disease pathogenesis. Independent experimental follow-up validating implicated taxa is rarely undertaken in this literature, so the phrase “clarifies how localised dysbiosis … drives systemic disease manifestation” should be read as a statistical association claim rather than a demonstrated causal mechanism.

#### 4.2.2 Recurrent Neural Networks

Recurrent Neural Networks (RNNs) are a specialised class of deep learning models designed to process sequential data. Unlike standard feedforward neural networks that treat inputs independently, RNNs feature internal feedback loops or memory mechanisms. This architectural design allows them to pass information from one step of a sequence to the next, maintaining a hidden state that retains historical context. Within microbiome research, more advanced variants like Long Short-Term Memory (LSTM) networks are frequently deployed because they overcome the standard RNN limitation of “forgetting” long-term dependencies. This capability makes them uniquely suited for biological data that is inherently ordered, whether it is the linear sequence of nucleotides in DNA/RNA or longitudinal data collected over time (Baranwal et al., 2022).

In the context of the host microbiome, RNNs have a transformative impact by shifting analysis from static snapshots to dynamic, predictive modelling. Because microbial ecosystems within a host are constantly changing due to diet, ageing, or medical interventions, RNNs are heavily utilised to predict future complex community behaviours and species-level abundance dynamics over longitudinal studies. Beyond temporal modelling, their impact extends to functional genomics and sequence-based tracking. RNNs are applied to multi-omics data, such as 16S rRNA gene or metagenomic shotgun sequencing, to identify microbial taxa, map host-microbiota interactions, and discover functional annotations like antibiotic resistance genes. By capturing the time-series trajectories and sequential constraints of microbial data, RNNs enable researchers to better understand how dysbiosis evolves, predict how a host’s microbiome will respond to therapies like probiotics or faecal microbiota transplants, and accurately diagnose health or disease states based on microbial progression (Baranwal et al., 2022).

It is worth flagging explicitly that, unlike the Random Forest, CNN, and autoencoder examples discussed elsewhere in this section, this subsection does not anchor its claims to a specific worked case study with quantitative performance metrics; the description above remains largely conceptual. This is itself informative about the state of the field: rigorously benchmarked, externally validated applications of RNN/LSTM architectures to human host-microbiome outcome prediction, as opposed to general biological sequence or genomic modelling, are comparatively scarce relative to CNN- and tree-based approaches. It should also be noted that vanilla RNNs remain vulnerable to vanishing or exploding gradients on long microbiome time series, which is precisely why gated architectures such as LSTM or GRU are required in practice rather than optional refinements (Baranwal et al., 2022); any claim of RNN utility in this space should therefore be understood as applying specifically to gated variants, not to recurrent architectures generally.

#### 4.2.3 Autoencoders

An autoencoder is a specialised class of unsupervised feedforward neural networks designed to learn compressed, efficient representations of high-dimensional data without relying on external labels. The core architecture consists of two symmetrical components: an encoder and a decoder. The encoder compresses the high-dimensional input vector into a lower-dimensional bottleneck layer, mathematically projecting the data into a reduced “latent space” that captures only the most essential, non-linear structural patterns. Conversely, the decoder accepts this latent representation and attempts to reconstruct the original high-dimensional input as accurately as possible. The network is trained by minimising reconstruction error, the discrepancy between the original input and the reconstructed output, which forces the bottleneck layer to discard noise and retain only the most critical, highly correlated features.

In the context of host-microbiome analysis, Dhakal et al. (2025) implemented an autoencoder framework to address the fundamental analytical challenges characteristic of microbial sequencing datasets. The authors utilised the encoder portion of the network to compress raw taxonomic profiles extracted from three distinct anatomical sites, the oral, lung, and gut compartments, into a unified, low-dimensional latent space. This process successfully preserved the intrinsic ecological characteristics and multi-compartment microbial interactions whilst eliminating statistical noise and structural redundancy.

The latent features extracted by the autoencoder were subsequently integrated with standard clinical parameters to train predictive models for host mortality risk. The interaction between the autoencoder-compressed microbial data and clinical features significantly enhanced predictive performance, yielding classification accuracies as high as 98% in lung-specific configurations. Furthermore, by evaluating the compartment-specific latent representations, the autoencoder framework revealed distinct predictive weights between body sites, demonstrating that the oral and lung microbiomes contained highly localised biological signals compared to the gut microbiome. Ultimately, the study establishes that autoencoders serve as an effective dimensionality-reduction pipeline, converting sparse, multi-compartment microbial counts into robust, clinically relevant biomarkers.

As with the other high-performing models discussed in this section, several caveats limit how far these results can be generalised. The 98% accuracy figure applies specifically to the lung-specific configuration; performance in the oral and gut compartments was not reported at the same level, and the corresponding manuscript does not describe evaluation on a held-out, temporally or institutionally independent patient cohort. Given the typically modest sample sizes of multi-compartment microbiome-clinical datasets, there is a meaningful risk that the reconstruction-plus-classification architecture is overfitting to compartment-specific noise rather than learning generalisable signal. It is also worth noting that the latent features, whilst useful for prediction, are not themselves directly biologically interpretable; describing them as “clinically relevant biomarkers” is premature without additional *post-hoc* analysis linking specific latent dimensions back to identifiable taxa or pathways.

#### 4.2.4 Transformer models

Transformer models represent a foundational shift in how artificial intelligence processes information. Historically, deep learning models like Recurrent Neural Networks (RNNs) analysed data sequentially, processing text word-by-word or genetic sequences base-by-base. This linear approach caused a computational bottleneck: it was slow, and the models frequently “forgot” information from the beginning of a long sequence by the time they reached the end.

Transformers overcame these limitations through two core innovations: the self-attention mechanism and parallel processing. Instead of reading linearly, a Transformer ingests an entire sequence of data simultaneously. The self-attention mechanism mathematically calculates the contextual relationship between every single element (or token) in a sequence and every other element, regardless of how far apart they are. For example, in the sentence, “The bank of the river had a steep slope,” a transformer instantly connects “bank” to “river” rather than “money” because it analyses the entire context at once. This capacity to capture long-range dependencies, combined with the ability to train on massive datasets through parallel processing, makes transformers uniquely powerful at finding hidden structures within complex data (Zhu et al., 2026).

In one of the reviews, it is explained that the power of transformers translates perfectly to host-microbiome research because biological structures like DNA, RNA, and proteins are essentially languages written by nature (Zhu et al., 2026). This account, however, is derived largely from the general natural language processing literature rather than from microbiome specific benchmarking studies, and the analogy between biological sequences and language, whilst conceptually appealing, has not yet translated into a body of rigorously validated, externally replicated transformer applications in human host microbiome research comparable to those available for CNNs or tree-based ensembles. Transformers are also notoriously data and compute hungry, typically requiring far larger training corpora than the hundreds to low thousands of samples available in most human microbiome cohorts; without large scale pretraining or transfer learning strategies, transformer models risk substantial overfitting on datasets of this size, a limitation the reviewed literature does not yet adequately address (Zhu et al., 2026).

## 5 AI-Driven predictive modelling of host–microbiome interactions

### 5.1 AI in disease prediction

The gut microbiome plays a central role in maintaining human health and influencing disease states (D'Urso and Broccolo, 2024). The emerging AI and integrative multi-omics approaches are advancing the field from descriptive profiling towards predictive modelling to enhance our understanding of microbial patterns associated with disease onset, progression, and treatment response with reasonable accuracy (Nguyen et al., 2022; Rozera et al., 2025). This dynamic intersection of AI and human health leverages ML algorithms to enable early diagnosis, personalised medicine, and tailored dietary or probiotic interventions (Kim et al., 2025). The innovative applications of AI are crucial for resolving the complexity and inherent variability of microbiome data across various conditions ranging from chronic gastrointestinal disorders to cancer (Zhou and Zhao, 2025).

#### 5.1.1 Inflammatory bowel disease

Inflammatory bowel disease (IBD), which includes Crohn’s disease and ulcerative colitis, is a severe intestinal disorder characterised by dysregulated immune responses (Taubenheim et al., 2025). However, our understanding of the role of disrupted host–microbial interactions in the pathology and aetiological mechanisms of IBD remains limited. AI-driven predictive modelling promises to enhance diagnostic accuracy and the effectiveness of IBD management. In the context of IBD classification, Tataru and David (2020) applied an embedding approach to derive latent microbiome properties from taxon co-occurrence patterns, resulting in improved classification performance, whereas a deep language model was trained in a self-supervised manner on large-scale unlabelled human gut microbiome datasets and subsequently applied to IBD prediction (Pope et al., 2025). A 2026 study of 429 patients with IBD used machine learning models incorporating baseline clinical, psychosocial, and lifestyle factors to predict IBD flares with the best-performing model achieving an AUC of 0.769; notably, the inclusion of psychosocial factors improved prediction compared with baseline clinical factors alone, whereas lifestyle variables provided limited additional predictive value (Okegunna et al., 2026).

A deep-learning model was developed to support IBD diagnosis by detecting and quantifying basal plasmacytosis, an early histological feature associated with IBD (Furlanello et al., 2025). Furthermore, AI-assisted analysis of pan-enteric video capsule endoscopy substantially reduced the pooled median review time to 3.2 min per patient, whilst maintaining 92–96% sensitivity and 90–93% specificity for Crohn’s disease detection (Brodersen et al., 2024). Moreover, the integration of CT enterography-derived radiomic features with machine-learning approaches has shown potential for differentiating ulcerative colitis from colonic Crohn’s disease (Wang et al., 2026), whilst MRI-based radiomics combined with clinical and imaging features showed potential for non-invasive identification of muscular alterations in Crohn’s disease, with improved diagnostic performance (Yu et al., 2025). In parallel, random forest models based on faecal microbes, faecal metabolites, and plasma metabolites distinguished high- from low-stress reactivity (SR) in UC, with AUCs of 0.81, 0.83, and 0.91, respectively; the resulting SR scores were associated with an increased risk of future clinical flares (Jacobs et al., 2024). A study from Marrakech compared logistic regression and Classification-and-Regression Tree (CART) models for predicting 12-month IBD relapse using clinical and biological variables. The traditional logistic regression model achieved an AUC of 0.71, with sensitivity 68%, and specificity 70%, whereas the CART model demonstrated superior predictive power with sensitivity 81%, specificity 77%, and an overall accuracy of 79% (Aouroud et al., 2026). With the development of robust models and standardised pipelines that generalise well, AI-enabled approaches could further advance personalised medicine for patients with IBD.

#### 5.1.2 Cancer

The role of AI integration in cancer research has become a promising technology for improving disease prediction and personalised treatment. According to the World Health Organisation (WHO), colorectal cancer (CRC) is the third most common cancer globally and remains a life-threatening malignancy. Waljee et al. (2022) review the potential significance of AI/ML algorithms in addressing colorectal cancer (CRC) in sub-Saharan Africa. The paper mainly highlights limitations in CRC screening and detection, and discusses two major AI applications: Multianalyte Assays with Algorithmic Analysis (MAAA) for early detection and, computer vision/pattern recognition approaches for histopathological diagnostics. The study urgently needs innovative AI-based screening approaches due to the rising incidence of cancer (Vargas-Santiago et al., 2025). Yamashita et al. (2021) developed the deep learning model MSINet to predict MSI status from H&E-stained whole-slide images of colorectal cancer patients. The model achieved a negative predictive value (NPV) of 93.7% on an external dataset and outperformed five experienced gastrointestinal pathologists in MSI prediction. Histopathological analysis plays an important role in CRC diagnosis, and CNN-based models have been widely applied for automated tissue-slide classification with high diagnostic accuracy (Thakur et al., 2020; Kuntz et al., 2021; Davri et al., 2022; Alam et al., 2022). Beyond tissue classification, these models can also facilitate the identification of clinically significant molecular patterns, such as microsatellite instability-high (MSI-H) and tumour microenvironment (TME) features.

AI/ML approaches have been extended beyond CRC to breast cancer, one of the most prevalent malignancies. Thereby, Hsieh et al. (2019) compared the performance of three AI models in predicting breast cancer risk amongst Taiwanese women with type 2 diabetes mellitus (T2DM) using logistic regression (LR), artificial neural networks (ANN), and random forests (RF). Using data from Taiwan’s National Health Insurance Research Database (NHIRD), a breast cancer risk prediction model was developed for T2DM patients between 2000 and 2012. The models were evaluated based on recall, accuracy, F1-score and AUC, with the RF model demonstrated superior predictive power in terms of AUC and several other performance metrics (Vargas-Santiago et al., 2025). These findings help to form the foundation for future research and the evolution of AI-based approaches in cancer diagnosis and treatment.

#### 5.1.3 Metabolic syndrome

Metabolic syndrome (MetS), including obesity, cardiovascular diseases (CVDs), stroke, and type 2 diabetes mellitus (T2DM), represents a major health concern. To predict obesity risk and associated metabolic disorders, ML models such as SVMs have been applied to genomic data. In particular, SVM models enriched with fat mass and obesity-associated (FTO) single-nucleotide polymorphisms (SNPs) have been used to classify the disease risk, achieving an AUC of 0.96 (Saikia and Kalita, 2025). Building upon disease risk stratification, the RF models show strong predictive performance. For instance, González-Martín et al. (2023) rely on transcriptomic and clinical data from 55 cohorts and report a prediction accuracy of 89.2% for insulin resistance (HOMA-IR > 2.5), with Gini importance analysis identifying SOCS3 and IRS2 as the key predictive biomarkers. Similarly, the XGBoost algorithm achieved a prediction of 93.1% in classifying metabolic components such as elevated waist circumference (>102 cm) and triglyceride levels (>150 mg/dL) by integrating a multi-omics dataset (Saikia and Kalita, 2025). In addition to identifying disease status, ML models are applied to long-term disease risk prediction. For example, Cai et al. (2023) employed a light gradient boosting machine (LightGBM) to predict the disease risk of developing MetS over a period of 10 years with an AUC of 0.774, whereas Wang et al. (2021) enabled an RF model to help achieve predictive accuracy of over 0.70 for MetS-associated individual biomarkers and 0.71 for overall risk assessment from complex datasets. Furthermore, in the application of AI beyond prediction to disease management, Bermingham et al. (2024) developed personalised dietary programmes using RF and regression models, significantly enhancing cardiometabolic health outcomes compared with conventional dietary recommendations.

### 5.2 AI in therapeutic intervention

The integration of AI into modern healthcare has evolved rapidly, enabling advances in diagnostics, drug discovery, personalised medicine, and operational efficiency. AI can predict how different probiotic strains will interact with the gut microbiome and influence human health by leveraging historical data and clinical trials. By analysing large-scale datasets from previous studies and clinical outcomes, AI can identify which probiotic strains are most effective for specific conditions or patient demographics (D'Urso and Broccolo, 2024). An AI model might predict that a certain combination of Lactobacillus and Bifidobacterium strains is particularly effective for managing irritable bowel syndrome (IBS) in adults, based on a comprehensive analysis of patient data and clinical outcomes (D'Urso and Broccolo, 2024). Dietary interventions, including personalised probiotic recommendations, based on individual microbiome profiles and health outcomes by incorporating data from genomic, metabolomic, and clinical sources. For a patient with a disrupted gut microbiome and specific metabolic abnormalities, an AI system might suggest a custom probiotic blend that targets the imbalances observed in their microbiome (D'Urso and Broccolo, 2024). For example, iProbiotics, a probiotic mining platform developed by Sun et al. (2022) that enables k-mer features within a support vector machine model, provides comprehensive genomic characterisation, functional features, and its applications. Meanwhile, for the identification of probiotics, Wu et al. (2024) introduced metaProbiotics, a computational framework that relies on language models. Pendulum Therapeutics is the first clinically validated probiotic formulation to support metabolic health. Beyond diagnosis and intervention, the Cox-net model has been widely used in time-series microbiome data and predicts patient survival risk by integrating personalised microbiome profiles and clinical measures (Zhou and Zhao, 2025). As a matter of fact, AI has also shown a prominent role in microbiome-based interventions, particularly in faecal microbiota transplantation (FMT) and phage therapy, both of which restore the microbiome and maintain homeostasis (Zhou and Zhao, 2025). FMT, an important strategy for restoring gut microbiota, applies to donor selection and microbial consortia to enhance the efficacy of personalised treatment selection. For instance, a recent clinical advancement shows that a phase III clinical trial of faecal microbiota live-jslm (RBL) for the treatment of recurrent Clostridioides difficile infection reported that the strategy not only restored microbial diversity but also achieved durable clinical remission through successful clonal engraftment of key microbial populations.

AI is advancing beyond imaging, creating new possibilities and insights into host microbiome interactions, with promising potential in disease risk prediction and therapeutics development, although its use in clinical translation is emerging.

## 6 Limitations and future aspects

Whilst integrative multi-omics and artificial intelligence have significantly advanced our understanding of host–microbiome interactions, many important directions remain to be explored. The convergence of integrative multi-omics technologies and artificial intelligence (AI) is rapidly reshaping host–microbiome research, providing unprecedented opportunities to uncover the molecular mechanisms underlying host-microbial interactions. Whilst significant progress has been made in characterising microbial communities and their functional contributions to host physiology, many aspects of host-microbiome crosstalk remain poorly understood. Future research must focus on comprehensive approaches that will enable a deeper understanding of microbial genes, metabolites, and signalling pathways, which collectively influence host health and disease.

The high cost of sample collection and data generation in multi-omics research is also a limitation, as it makes it difficult for individual studies to detect small but meaningful associations. It is necessary to build shared repositories for microbiome multi-omics data, which would be highly beneficial in overcoming this limitation, whilst careful attention must be paid to differences in data-processing methods and batch effects across studies to ensure results are comparable and reliable.

Another key area of development is predictive modelling of microbiome-derived metabolites, including short-chain fatty acids, bile acids, and tryptophan metabolites, which are central mediators of metabolic crosstalk between hosts and microbial communities. Integrating metabolomics with AI-driven network analysis may enable personalised prediction of metabolic responses, disease susceptibility, and therapeutic outcomes. Such approaches could facilitate the development of precision medicine strategies tailored to an individual’s microbiome composition and functional profile.

Recent advances in foundation models and large-scale AI architectures also offer exciting possibilities for microbiome research. By integrating heterogeneous datasets across different biological scales, these models may generate comprehensive representations of host–microbiome interactions and improve the prediction of complex phenotypes. Furthermore, the incorporation of biological knowledge, such as metabolic pathways, microbial interaction networks, and host signalling cascades, into AI frameworks could enhance both predictive performance and biological interpretability.

## 7 Conclusion

The emerging AI and omics technologies together enable a much deeper understanding of the microbiome and its impact on human health. It is crucial to understand the intricate relationships between host-microbiota because it helps to predict diseases, tailor treatments, and gain more precise knowledge of disease dynamics. This review offers a comprehensive exploration of the use of integrated multi-omics and advanced tools in AI/ML models, which could find applications across various domains. Despite this practical feasibility, several limitations, such as model generalisation, data interpretability, and methodological standardisation, remain significant hurdles to overcome before AI-driven model insights can be translated into clinical practice. To overcome these challenges, the development of robust validation models is essential, as such analytical frameworks hold significant potential to facilitate more accurate diagnostic approaches, predictive nutrition, and therapeutic strategies.
